# On the Outside Looking In: A Global Doula Response to COVID-19

**DOI:** 10.3389/fsoc.2021.613978

**Published:** 2021-02-19

**Authors:** Julie Johnson Searcy, Angela N. Castañeda

**Affiliations:** ^1^ Department of History and Anthropology, Butler University, Indianapolis, IN, United States; ^2^ Department of Sociology and Anthropology, DePauw University, Greencastle, IN, United States

**Keywords:** doulas, birth, COVID-19, reproduction, pandemic

## Abstract

From around the world, doulas report the impact of new COVID-19 restrictions on their ability to provide continuous emotional, physical, and informational support to pregnant people and their families. In a qualitative survey conducted in March and April 2020, we heard from over 500 doulas in 24 countries. Doulas practicing across the world revealed rapid changes to hospital policies. Even accounting for different public health responses across countries, the doulas in our study pointed to one common theme - their absence at births and the subsequent need to support birthing people virtually. In a follow-up survey and in interviews we conducted in July, we reconnected with doulas from our initial study to track their access to institutional birthing spaces. As countries experienced the effects of “flattening the curve,” we found that doulas were still not considered “essential” workers and the majority could not attend births. Our research shows that doulas have ambiguous feelings about the efficacy of virtual support, that they raise concerns about the long-term impact of COVID on their profession and that they are concerned about mistreatment and obstetric violence as birthing people enter hospitals alone.

## Introduction and Methodology: A Changing Birth Landscape

Months into the global COVID-19 pandemic, communities across the globe continue to adapt to new rules and regulations surrounding birth culture. In particular, data gathered in the United States highlights how quickly policy changes have shifted for pregnant people, families and providers (Davis-Floyd et al., 2020). This includes the global community of birth workers known as doulas, who herein report the impact of new COVID-19 restrictions on their ability to provide continuous emotional, physical, and informational support to pregnant people and their families (Castañeda and Johnson Searcy., 2020).

This research focuses on the experiences of doulas drawn from a qualitative survey conducted in March and april of 2020, which collected over 500 responses from doulas in 23 countries. The survey included a mix of fixed-choice and open-ended questions, including asking doulas about the spaces they practiced in, if they worked in urban, rural or suburban areas, and an open-ended question about which area of the world they practiced in. The other open-ended questions in the survey asked doulas to reflect on and describe different aspects of their work in relation to COVID-19. The survey also included the option of indicating interest in a follow-up interview. We posted this survey on 24 doula Facebook groups, including general groups (Love What You Doula, Doula Talk); geographic-specific groups (i.e., Doulas of CA, Doula Connect - FL) and demographic specific groups (Doulas and Midwives of Color, Doula Latina, Queer Doula Network). We also conducted internet searches for doula organizations and collectives in countries outside of the US and sent emails explaining and attaching the survey and asking for the survey to be distributed to interested doulas.

We received response from 515 doulas. 373 of these responses came from doulas in the United States from 42 of the 50 states. The remaining survey responses came from 22 different countries (South Africa, Canada, New Zealand, Australia, Germany, Italy, Finland, Hungary, Portugal, Ireland, Sweden, Denmark, Japan, United Kingdom, Taiwan, Israel, Peru, India, Dubai, Mexico, Argentina, Bolivia). The responses we received from each of these countries varied from 80 to a handful, making it difficult to create a statistically significant comparative analysis. In addition, not all respondents indicated their geographic location. To analyze these qualitative responses, we separately coded each survey response, line by line, looking for themes and concepts and prioritizing the language doulas used to describe their work during this pandemic. We then compared and triangulated the themes for analysis. We conducted a follow-up qualitative survey and interviews in July 2020 to see how doulas saw the pandemic continuing to affect their ability to “mother the mother.” Response to this survey was much smaller and included 52 doulas from the US, Canada, South Africa, Argentina, Spain, Japan and New Zealand. Of these respondents, 43 of the respondents had taken our first survey. We repeated the same coding process with this survey and then compared themes to the first survey. What became apparent while coding were the similar experiences, fears and challenges doulas described both in the early days of the pandemic and a few months further in. The follow-up survey confirmed and elaborated on themes that were present in the first survey. While we recognize the variation in context for many of the responding doulas and the limitations of the survey to capture all of the differences among regions of the world, we were struck by the commonality of the themes doulas described of disruption and rupture to the very nature of their work.

Doulas practicing across the world reported rapid changes in hospital policies with the onset of COVID-19. Despite the variation in public health strategies reported from different countries, the doulas in our studies were most concerned about their absence at births. The overwhelming and near-universal restrictions on doula support in hospitals, and even in some birth centers and at home births, spanned six continents and held across the time span between our two surveys—and aligned with a sharp increase in virtual doula-ing. Comparing each country lies outside the scope of this article; instead we focus here on the common themes and patterns doulas describe about the continuing impact of COVID-19 restrictions on these birth workers. We found that doulas raised concerns around three themes: 1) the efficacy (or lack thereof) of virtual care; 2) the impacts of pandemic restrictions on doulas as a profession coalescing around the politics of “*The essential*”; and 3) the concerns about increasing obstetric violence without a birth partner to bear witness. Our research asks us to consider how doulas’ experiences around the globe can help us understand the power of a pandemic to influence birth culture.

## New Hospital Restrictions and Policy Challenges: The Politics of “The Essential”

COVID-19 ushered in new hospital restrictions across the globe leading to new configurations about who counted as “essential” at a birth. In the context of labor and birth, essential workers became defined by changing hospital protocols, health provider preferences, and new stay-at-home orders from local and national governments (Castañeda and Johnson Searcy 2020). Doulas initially reported a dizzying flurry of shifting policies that ultimately kept most doulas out of the hospital. The majority of respondents commented on changes to hospital protocols, as with this doula from Germany:

“Hospital policies are constantly changing as far as birth support. All hospitals have a maximum of one accompanying person. In some places, the partner must be from the same household (so doulas not allowed). Some allow this partner to be present for (the) entire labor (while) some just for the pushing phase. Most hospitals allow very limited postpartum accompaniment. Most hospitals are releasing newborn/mom as early as possible.”

Doulas pointed out how erratic the policies were and that they differed among hospitals. A doula in Portugal described it this way, “Hospital policies are totally different. I cannot enter, and in some cases, women cannot have their phones with them. They have to go alone, so we can’t even talk with the father.” If any labor support was allowed, laboring people were often forced to choose only one person. A doula in the Ireland described this choice:

“I have lost some clients who were interested in hiring a doula as they don’t want to have to choose between their partner or doula for labor and also finances have been affected so maybe now paying for a doula is not affordable any longer.”

Doulas watched as hospital policies asked birthing people to choose only one support person. Many doulas told us it wasn’t a fair choice for families to make because they understood the importance of the partner being there. A South African doula explained this dilemma:

“My Doula business has been declared a Non-Essential role in the Birth Team. The Hospital refused my assistance in the Labor Ward so I had to go back home. The Mother had to choose between me and her husband as if we play the same role at the Birth..Oh, she eventually had to undergo an “emergency” Caesarean operation.”

In this example, the doula clearly points out how hospital policies do not fully take into account the difference *and* mutual relationship between a doula and partner. This neglects how both a doula and partner work together with a laboring person. Doulas draw on a partner's intimate knowledge of the laboring person and thus the partner becomes an integral source of help to the doula to best assist the laboring person.

Doulas have always faced questions about the economic worth of what they do. This pandemic has heightened that issue as people consider whether virtual doula work is worth the cost. Doulas have long had to work to convince people that a supportive witness at birth can shift outcomes (Kayne et al., 2001; Hunter 2012; Bohren et al., 2019). Doulas have come up against barriers in their work in clinical spaces and scholars have written about the liminal space they occupy (Kayne et al., 2001; Everson and Cheney, 2015). Some doulas argue that their presence is necessary because a biomedical model of birth does not attend to (or often acknowledge) people’s emotional and physical needs during labor. Because a biomedical model sees birth from a narrow framework, doulas have long had to fight for their place at the side of birthing people. And it is only very recently (in the last 5 years) that we find widespread institutional acknowledgment of the importance of a birth partner (WHO). COVID-19 policies that keep doulas away from people in labor and necessitate virtual doula support make doulas afraid that they will be re-framed as unimportant—as they originally were—and that this will impact women in profound ways. Doulas moved online as pandemic policies required, but worried about the fallout for those giving birth. {moved from *Economic Instability* per the reviewer’s recommendation}

### From Policy to Personal Obstacles

Doulas faced challenges beyond entering hospitals. The ability to travel and attend to clients in their homes depended on whether a doula either self-identified as “essential” or had access to supportive documentation or support from a health worker. In South Africa, a doula shared, “I am armed with a copy of my ID and Permit when I go on the road to visit my postnatal and Antenatal clients at their homes.” Another doula reported, “Officially no private or government hospitals allow doulas in South Africa any longer. Some doctors have given permission, and mothers with no birth partner may do so but the new doula restrictions are strongly enforced.” Another issue was whether there was even local transportation available, as shared in this case from Peru:

“My work is difficult now especially due to transit being restricted. I was just a doula at a birth two days ago in the local public hospital, and I had to return from there walking to my house! I had to have permission to travel since there is a curfew from 6pm every day.”

Doulas often managed to work around these challenges. They talked about which training organizations could certify them, with as little money and time as possible, in order to procure certification that would allow them in the hospitals. Some had influential clients helping them secure permission to travel. And despite being shut out from hospital births, doulas still found ways to continue their care, for example in the form of “care-box drop offs with herbs and ingredients for Ayurvedic recovery recipes” (South Africa).

Even as doulas pivoted to adjust to the ways in which the pandemic was changing the conditions of their work, they also expressed concern for their clients and for their own personal challenges that working as a doula now entailed. One doula in Canada told us, “I am navigating my relationship with my profession in a fundamentally different way. This is leading to both fulfillment and compassion fatigue. Birth work could use some shaking up, but the environment of fear is becoming rampant and toxic leaving (particularly students and new parents) in a lot of uncertainty.”

Tracing doulas’ responses to the pandemic through its first six months, we heard multiple patterns of concern. Alongside the emotional stress doulas carry as they work to provide intimate labor to their clients, they must simultaneously navigate near-impossible work-life constraints. From Germany, a doula summarized the situation: “It's a huge challenge. I have less clients, less income, and it's hard to have a quiet space at home while I myself have two young children who are also stuck at home.” A doula in the US told us, “When I’m home I’m less available even if I’m physically present because I’m preoccupied. Previously I would leave to support. Now I support from the couch, which looks different to [my] children who can’t understand the circumstances.” And from Mexico a doula shared, “Doulas are agents of security and serenity for our clients but now, we are tasked with this in all new levels for our own families as well.” Doulas also worried about moving their support for people in labor to a virtual platform; this was often their only option, given the global policies around attending births in most places.

## Virtual Doulas

Doula practice is rooted in accepting change, remaining flexible and adapting to new scenarios (Hunter 2012; Castañeda and Johnson Searcy., 2020). Birth is different with every client; comfort techniques that work for one woman may not work for another. To be effective, a doula must learn to read what an individual in labor needs. For example, doulas often maneuver through relational dynamics – mothers-in-law who aren’t welcomed by the birthing person or tension between partners or spouses. Because doulas most often follow women to their chosen birth location, doulas may attend births in multiple places: different hospitals, birth centers, and homes. Adaptation and flexibility are necessary for navigating each of these spaces. The degree to which doulas are required to adapt and change their practice drastically increased during COVID. Doulas reported that the pandemic necessitated a quick transition online. Doulas told us that they used a variety of platforms including Zoom, Facetime, Skype, Whatsapp, and Slack. One doula described how virtual work was transforming doula care. She said, “I shifted to fully virtual for hospital clients, a completely new experience for me. I am unable to physically be with my clients for prenatal visits, birth, or postpartum. Losing this aspect of my work is heartbreaking. Of course, any new consultations are taking place virtually as well” (USA). Doulas grieved the loss of physical presence and understood virtual support to require something very different from them. A Canadian doula told us, “All of it is online. It was not as simple as just taking what we used to do in putting it online. It is a completely different service now.” Doulas had to rethink their practices of support and presence and refashion them for virtual formats. Doulas relayed how they made Whatsapp calls, sent voice messages and birth affirmations, and provided flash drives with hypnobirthing music. In our follow-up responses, it was clear that doulas were continuing to rely on virtual support to access their clients. One South African doula shares her approach:

“I explain in depth what virtual support looks and feels like. I help set up the technology necessary and practice ahead of time that we can get good camera angles etc. and I “attend” the birth pretty much as if I were there in person. I watch and listen and offer suggestions to the partner. If no partner is allowed, I have the birthing person put on earphones to hear me supporting and encouraging etc.”

Yet even virtual presence at a birth can be broken. One Canadian doula told us that she was present via a laptop that had been set up so she could see the room and talk with the laboring woman and her partner. When the woman reached the second stage and was ready to push, the doctor entered the room and shut the computer screen, rendering this doula useless in those important moments.

Because attending a birth virtually can be difficult, doulas also now practice with much more focus on prenatal care. The emphasis on new ways of working often involved greater participation from a pregnant person’s partner. Doulas managed their absence during birth by emphasizing the importance of education and preparation. Some doulas reported success with this approach:

“I’ve realized that preparing my clients ahead of time and suggesting techniques to use has proven much more useful than I could have known. A client recently delivered her baby in the driveway on the way to the hospital because she was so focused [from prenatal preparation], she didn’t realize that she was in labor until her waters broke, and from that point, she gave birth within 40 min (South Africa).”

Doulas described examples of preparing partners as stand-in labor support when doulas are unable to attend a birth. A New Zealand doula recounts how her new practice involves, “Directing instruction specifically to the birth partner and encouraging the couple to actively practice techniques of massage, breathing, relaxation, and affirmations.” And in Canada, a doula detailed the changes involved in becoming a virtual doula:

“We used to have two prenatal sessions and then in person support. We now have five prenatal sessions because we need to train the couple in a lot more things when it comes to preparing for Birth and bringing home a baby. We are basically teaching the partner to be a Doula and then guide them periodically throughout the labor experience to help the partner recall the information and techniques.”

Despite the stark differences in virtual vs. in-person doula care, some doulas were adamant about their ability to “hold space” for intimate care, as explained by a doula from South Africa: “Holding space does not need to be done in person. Being available to clients and encouraging and guiding them to be self-empowered is now my focus. Preparation prior to birth is of utmost importance.” Some doulas worked to bring in personal practices to create and “hold space” virtually, focusing on their own breathing, ensuring a quiet, calm space on their end of a virtual connection, hoping that calmness would transfer to their client in the hospital.

### Efficacy of Virtual Doula Support

While our surveys confirm that the majority of doulas made the switch to virtual labor support, the efficacy of this new approach remains unclear. As a doula in Italy wrote, “I'm trying with the online but it is not the same … ” A German doula noted the important emotional labor that her work entailed and said, “It’s much more difficult to engage in heartfelt conversations. It feels like the virtual platform transforms the prenatal sessions into more of a lecture or informational class rather than a collective, emotional discussion.” Other doulas felt that the virtual medium itself was not congruent with helping a person in labor. They were concerned about interjecting more technology into an already heavily technology-driven hospital. For example, one doula from South Africa shared, “No, I don’t do virtual. I only trust the amazing womanly body that knows how to birth and teach women to listen to their bodies. Not a new technique but quite forgotten.” And another South African doula explained, “I do not enjoy virtual support as I find it’s too much neocortex stimuli for the birthing person, and I find technology around internet connection expensive and unreliable.” Doulas in the US drew attention to equity issues, concerned that people who most needed the support would not have the kind of access required to make virtual doula work effective (Searcy and Castañeda 2020).

All of the challenges around virtual doula work resulted in some doulas reporting to us that they outright did not like it. One Canadian doula who attended a birth virtually felt she could not effectively participate or provide support. She told us, “I felt like I was just a fly on the wall.” Another doula in Japan was much more explicit in her dislike for working virtually: “I actually hate virtual birth work. It is not the same. I now firmly believe doulas improve outcomes because they are there in person. It’s been very frustrating for me and very lonely and scary for my clients.” Doulas lament the restrictions on their ability to attend births and help new-formed families, with one doula from India affirming, “This is a field you can't [have] without touch.” These doulas saw their presence as a critical component of the support they provide women; research on birth partners confirms the benefits of a physically present birth attendant and shows that the well-known “doula effect” of shortening length of labor, minimizing interventions, and increasing psychological satisfaction only holds if the doula is indeed physically present throughout the labor and birth (Sauls, 2002; Gruber et al., 2013; Steel et al., 2015).

### Economic Instability

Doulas’ pivot to virtual support required them to navigate uncharted territory and to deal with increasing financial stress. Doulas repeatedly pointed to the COVID-19 policies as the cause of the new economic instability they were experiencing. As many hospital policies precluded doulas from being present at births, they could no longer charge for the support they offered in person. One doula from Portugal described the drastic changes to her economic identity as “the fragility of my income.” As lockdown orders went into effect and hospital policies changed, doulas dealt with canceled contracts or scrambled to rewrite contracts and refund payments for incomplete services. We heard repeatedly about increased stress, as for this United Kingdom doula: “Lots of stress and worries about how this will impact financially and whether I will be able to provide the services I have already been hired for.” A South African a doula shared, “Clients are more anxious and need more emotional support, my income has diminished just about completely and I need to refigure how to charge clients in a climate like this.” Doulas struggled to translate the value of their virtual services to current and potential clients, saying “My clients’ income has reduced, and now they are reluctant to pay [for] even online support” (South Africa). A doula in Canada noted that “a significant aspect of my approach to support was physical, however, I'm exploring the other aspects of my support tool kit—information gathering, spiritual support and emotional guidance. Navigating payment has also significantly shifted and I find myself doing significantly more unpaid work.” If doula services were categorized as a “luxury” before COVID-19, they most definitely would be after the start of this pandemic, as people all over the world saw their incomes negatively impacted: “Financially it will take a long time to recover from COVID-19 as people’s income has been affected and we are a ‘luxury’ spend” (New Zealand). Here again we see the debate over whether doulas are essential workers or not, which we have called “the politics of the essential” at play: this New Zealand doula sees doulas as non-essential “luxuries.”

## Impacts on the Doula Profession and the Politics of “Essential” Workers

As governments across the globe worked to contain the COVID-19 pandemic, including implementing new stay-at-home orders for non-essential personnel, doulas have struggled to find a sense of belonging in the rapidly changing birth landscape. A consistent theme heard across doula responses was frustration with how these new restrictions limited their work, most often attributed to doulas finding themselves forced into a debate about their role as essential workers. One doula from New Zealand put this succinctly when she said, “We were totally disregarded as ‘essential’ which was heartbreaking.” Another doula from South Africa reported:

“We have been fighting very hard to show doulas’ worth here, and in a second called us "non-essential" and closed all the doors to us again. This shows me that any change or gain we made was not based on merit or the research that showed all the benefits of a doula, and so it would never have been a lasting change.”

Pandemic policies that restricted doulas’ access to hospitals illuminated a much longer and larger struggle doulas have had globally as they try to assert the importance of the services they provide to birthing people. Doulas, like the South African one quoted above, felt the sting of being labeled “non-essential” during the pandemic, in part because that is how doulas have often been seen in biomedical spaces (Gilliland, 2002; Norman and Katz Rothman, 2007; Stevens et al., 2011; Neel et al., 2019). Doulas often occupy a liminal space, moving between multiple worlds, crossing boundaries (Everson and Cheney 2015; Horstman et al., 2017). Doulas’ presence in hospitals often hinges on carefully cultivated relationships with local hospitals, as doulas seek to demonstrate the importance of their role during labor and birth. With no official recognition or role within the biomedical system, doulas have long worked to assert themselves as “essential” (Norman and Katz Rothman, 2007; Roth et al., 2016; World Health Organization, 2019). Being rendered “non-essential” during a pandemic heightens the tension on the tightrope doulas already walk. A doula in Canada captured this tension when she told us, “It has been incredibly stressful navigating constant changes in a system that doesn’t recognize that we exist—no official public health body has named doulas as a profession/industry and therefore have not provided clear instructions to us.” As this doula points out, the pandemic amplified doulas tenuous and often liminal status. Despite doulas efforts to professionalize, as evidenced by the many doula certifying and continuing education options now available to them, there is no outside institutional consensus or recognition of doulas professional role (National Academies of Science Engineering and medicine, 2020) making their status as essential always up for debate.

The void in clear direction created by the rapid change in hospital protocols left many doulas scrambling to make sense of their role at births and revealed deeper intrinsic debates among doulas about their role. South African doulas demonstrated the way this rapid change foregrounded the debate within the doula community about where doulas belong and what world they are a part of. One doula from South Africa wrote:

“Some doulas are willing to support clients at their homes, before going to the hospital, during our lockdown. I wasn’t, so that is why a client transferred to another doula who was willing to come to her home. There has been a huge debate about whether doulas are healthcare workers and whether they are essential. The “permission” we got to travel during the lockdown, in my opinion doesn’t really give us permission, as we are deemed not essential and not medical persons. It’s a sad state of affairs for doulas, but some are not willing to accept this as the current state.”

This resulted in a split across doula communities, with some identifying as essential workers and wanting to be on the front lines, while others saw their scope outside of “essential worker” parameters and agreed to being sidelined during the pandemic. Another doula from South Africa found this divide to be one of the biggest challenges:

“Convincing other doulas that they need to do the socially responsible thing and NOT attend births or client visits in person, that’s what’s most challenging. I run the local doula organization and this has been by far the biggest challenge. Despite legal definitions and new laws, Doulas continue to fight the system and attempt to find loopholes, committing fraud to do it.”

This doula refers to reports of doulas in South African (and other places including the US) who used loopholes in hospital pandemic policy to get into hospitals as fraud. From her perspective, doulas who hastily put together paperwork that could appear as some kind of certification and thus grant them entry into a hospital, was fraud and beyond the scope of practice doulas should undertake. Another South African doula also saw the decision to not provide in-person doula care as the right thing to do:

“I applaud hospitals for both realizing the value of Doulas but also being aware of their limitations during a pandemic. Contrary to popular belief, doulas are NOT medical professionals and as such, although a wonderful part of a birthing team, they are not essential healthcare workers. Doulas are not trained to prevent the spread of any virus and while it is infinitely sad to all of us (and our clients!) that we may not attend births anymore as we are not an essential service, it is the socially responsible thing to do!”

Thus, we can see that some doulas felt strongly that it was in their best interest and that of their clients to act according to public health rules and recommendations and so were in conflict with those who felt their role was essential at births. Some doulas felt that complying with public health policies that mandated doulas as “nonessential” was a “sad state of affairs” but the necessary reality. Other doulas felt that attending births was crucial to demonstrating the critical nature of a doula’s role. In the doula community, the debate over who is “essential” demonstrates the political stakes; doulas who wanted to claim “essential” worker status hoped to do so as part of an ongoing effort to secure their role in reproductive care.

Many doulas also expressed concern about how current restrictions on doula presence would shape their profession moving forward. As one doula in Germany pointed out, “It is a difficult time that is testing my ability to do this work. The scariest part is that after the full ‘crisis’ is over, will hospitals continue to implement these restrictions and limitations on birthing support?” The uncertainty with the future was coupled with current frustration and grief due to changing hospital protocols, as a doula in South Africa lamented, “I feel we have worked so hard to be known and seen by medical staff, we’ve been always walking on a slippery slide and now we have been pushed down when women need our support more than ever.” Doulas work to stay in the moment at birth, but the uncertainty of their future was repeatedly cited as occupying the forefront of their concerns. “It's going to be hard picking up the pieces when this is finally over. It's already been hard to build up trust and build relationships with doctors and hospitals in South Africa. Doulas are losing income and clients lose support.” The global trend of classifying doulas as non-essential continues to keep doulas concerned about their professional standing in clinical settings.

## Concerns About Obstetric Violence

Doulas’ concern about losing their place at the table of reproductive care was in part because of their fears that mistreatment and obstetric violence would escalate without their presence. We heard this theme come up again and again as doulas around the world reflected on the ongoing pandemic and restrictions. Doulas saw hospital protocols that required birthing people to be alone without anyone accompanying them as “a basic human right being taken away from parents” (South Africa). Another doula from South Africa said, “I am unable to support women in the government hospitals who are from township areas and have no support at all. They are alone and don't know what is happening to them.” A doula in Ireland shared, “Hospitals have a one birth partner only restriction and some hospitals have banned all partners and mums have to go through labor alone,” and from Hungary, “Doulas were excluded first, now fathers too. This means more obstetric violence and more vulnerable women.” Other doulas in our study also expressed concern about increasing rates of obstetric violence occurring due to COVID-19 restrictions. A doula in Argentina affirmed, “Everything that happened before the pandemic in a pandemic worsens, grows bigger. Obstetric violence became more severe,” as well as from Spain where “birth has involved emotional and physical obstetric violence.” Doulas in the US were concerned about marginalized populations, describing how pandemic policies meant that “There is a lack of personal contact I would normally be allowed to provide. Basic human rights are being taken away and women are afraid. I work with young moms who are being talked into procedures they previously didn’t want.” Another US doula told us that the changes had a significant impact on her clients, whom, she felt, were at increased risk without doula support:

“My clients are young, single, clinic patients who are typically women of color. Their care within the hospital is not given with dignity and respect. I have seen it with my own eyes. Without having a doula there for support, these girls are at the mercy of the doctors and nurses, as they don’t feel empowered enough to speak up or question anything. It saddens me greatly, as this was why I got into doula-work.”

Doulas saw themselves as witnesses to institutional mistreatment and violence and as providers of support to people who received such treatment. They also stressed that their presence at birth as witnesses is a deterrent to obstetric violence, disrespect, and abuse.

With the incorporation of virtual technology, doulas are now witnessing obstetric violence through a new lens. We heard this perspective from a doula in Japan, “My clients are treated much differently when I’m not in the room. I never fully understood this until virtual work.” From Canada to South Africa, doulas reported examples of obstetric violence, including clients “overlooked due to shortages,” as well as experiencing “lack of consent” and “fear mongering being used to get them to do a cesarean.” In particular, South African doulas affirmed, “I feel as if women are now treated even harsher than before. More like a production line at hospitals now,” and “Also, a lot of obstetricians are convincing moms of the ridiculous idea that C-sections are the safest option in this time.” The results of these stressful experiences are visible during and after birth, as one doula reported, “I see stress taking over and partners worried during labor and postpartum” (South Africa). Without the power of witnessing and of supportive touch, whether in birth or postpartum with breastfeeding help, doulas worry about the damage being done to new families. This situation, described by one doula as “a recipe for postpartum depression” (South Africa) is leaving doulas to work virtually with new parents who are scared and concerned. A US doula confirmed:

“My clients are terrified to not have a doula with them, and be stuck using virtual platforms. I have experienced obstetric violence, mistreatment, emotional abuse, lack of informed consent over virtual platforms. The presence of a doula alone causes an increase in true informed consent sessions, and therefore better outcomes. So with doulas being limited to virtual platforms, a few of my clients have experienced coercion and negative outcomes. One of them was pretty traumatized by the treatment she received, which means I am now watching her closely for postpartum depression. With the first postpartum visit usually being around 6 weeks, I now feel it is my duty to go above and beyond to make sure she is safe.”

Preliminary research suggests that COVID’s impact on postpartum depression is real (Davenport et al., 2020), and additional long-term studies will reveal the consequences of COVID policies that restrict support for birthing people.

## Doulas and the Home Birth Increase

While many doulas described feeling “defeated” or “hopeless” with the pandemic situation, we also found areas of positive engagement, including more interest in home birth. Doulas reported a sharp increase in requests for home birth and information on midwifery models of care. As a South African doula explained:

“This situation seems to have low risk birthing families to contact, discuss and look to midwife led facilities. Home births are being considered more also. This pleases me so much as it’s directing birth back to where it belongs—with midwives and midwifery led units and at home.”

This situation has also changed how some doulas serve clients by expanding their scope of practice. For example, “I no longer volunteer at the local government hospital maternity ward. I work with a midwife and we are snowed under with requests to support our midwife led birth unit, and we now do home births for women who can’t travel across provincial borders” (South Africa). Another South African doula wrote:

“At the midwifery unit we now do home births which we didn’t do before. We have taken on another midwife and are looking for another doula. We are busier than ever before. I have not been to a government hospital for 4 weeks and no longer can support families at private hospitals. The main private hospitals have officially declared “No Doulas.””

According to Davis-Floyd et al., (2020), as COVID swept the country, numbers of US childbearers made a rapid switch to home birth to flee hospital contagion and also to avoid having to labor alone or to be forced to choose between partner and doula. One of our a US doula respondents stressed the latter reason as she shared her experience with the rise in home births:

“The number of home births in our area went up astronomically in the past few months. Women who had never considered a home birth before, or had considered it but never made the leap, switched to home at the last minute. I was a part of 4 of these births in the past two months, and every one of them gave the same reason for switching—not that they were afraid of COVID exposure, but that they didn't want to be refused doula support in the hospital. Wow!! I think that speaks to the importance of doulas at births, and how badly women want them there.”

Faced with mounting uncertainties both with the virus and changing hospital policies, pregnant people turned to doulas for help navigating the possibilities of home birth and midwifery models of care.

## Conclusion: The Need to Increase Doula Care

Research with doulas shows the power of the pandemic to intensify and homogenize biomedical, technocratic models of birth (Davis-Floyd 2003). As a South African doula described, “It reveals that psychological, practical, and emotional care during birth is still not considered a priority.” We recognize the need for doulas to have training in infectious disease as well as proper personal protective equipment in order to serve their clients and safeguard other medical personnel. As one doula shared, “In uncertain times, doula care is even more important for mothers. If both parties desire, I believe there are safe ways to provide in person care for healthy families and doulas” (United Arab Emirates). Working within these parameters in person, doulas increase emotional and social support for laboring people as well as alleviate some of the stress of caring for them for hospital management. This sentiment was echoed by a South African doula:

“While I understand that doulas are not medical professionals, and respect the need for distance and protection, we make essential differences to the birth experience for parents. Our responsible presence at births would make it so much easier, not only for the parents, but also the entire medical team during this time of fear and uncertainty.”

Many doulas called on medical institutions to recognize and legitimize the support they bring to birth, and to code them as truly “essential.”

As countries around the world continue to deal with varying rates of COVID-19 infections, from “flattening the curve” to second or third waves, many unanswered questions remain. This uncertainty is reflected in our most recent responses from international doulas. While some international respondents reported affirmatively that doulas were indeed readmitted as birth workers in hospitals, they were in the minority, as many others confirmed the continued exclusion of doulas. The answers to our questions were not bound by national borders, as we gathered differing responses from doulas within the same national territory and similar responses from around the globe. These responses only further reinforce our understanding that this virus knows no political borders—national, regional or local. Instead, what we can affirm from our international responses is that doulas around the world are continuing to support laboring people, sometimes in person and other times virtually, and that their commitment to a holistic form of care is rooted in their belief that the liminal moments of birth are both transformational and lasting. Doulas are expanding and increasing their intimate labor across platforms as they move online to continue to invite others to “breathe with me.” A doula in Australia put it this way:

“I really hope that rules and regulations recognize the important work doulas do in this time. I feel like they are the ones stepping up right now and trying to fill all the voids left in this strange time. Creating online mothers’ groups, connecting new mums up with each other so they don’t feel so alone, and being there consistently to talk to expecting mums. Please don’t forget them and please acknowledge what the doula network is doing right now.”

Now more than ever we need to recognize and support the essential work that doulas do, as well as invest in strategies that increase access to doula care for women worldwide in sustainable ways.

## Data Availability

The datasets presented in this article are not readily available because Authors have made anonymous all qualitative data from respondents. Requests to access the datasets should be directed to jsearcy@butler.edu.
